# Q Fever Outbreak among Workers at a Waste-Sorting Plant

**DOI:** 10.1371/journal.pone.0138817

**Published:** 2015-09-23

**Authors:** Eva Alonso, Idoia Lopez-Etxaniz, Ana Hurtado, Paloma Liendo, Felix Urbaneja, Inmaculada Aspiritxaga, Jose Ignacio Olaizola, Alvaro Piñero, Iñaki Arrazola, Jesús F. Barandika, Silvia Hernáez, Nerea Muniozguren, Ana L. García- Pérez

**Affiliations:** 1 Department of Epidemiology, Subdirección de Salud Pública de Bizkaia, Gobierno Vasco, Bilbao, Bizkaia, Spain; 2 Department of Epidemiology, OSALAN- Instituto Vasco de Seguridad y Salud Laborales, Barakaldo, Bizkaia, Spain; 3 Department of Animal Health, NEIKER- Instituto Vasco de Investigación y Desarrollo Agrario, Derio, Bizkaia, Spain; 4 Department of Microbiology, Hospital Universitario de Basurto, Bilbao, Bizkaia, Spain; 5 Department of Agriculture, Diputación Foral de Bizkaia, Bilbao, Bizkaia, Spain; Texas A&M Health Science Center, UNITED STATES

## Abstract

An outbreak of Q fever occurred in February–April 2014 among workers at a waste-sorting plant in Bilbao (Spain). The outbreak affected 58.5% of investigated employees, 47.2% as confirmed cases (PCR and/or serology) and 11.3% as probable cases (symptoms without laboratory confirmation). Only employees who had no-access to the waste processing areas of the plant were not affected and incidence of infection was significantly higher among workers not using respiratory protection masks. Detection by qPCR of *Coxiella burnetii* in dust collected from surfaces of the plant facilities confirmed exposure of workers inside the plant. Animal remains sporadically detected among the residues received for waste-sorting were the most probable source of infection. After cleaning and disinfection, all environmental samples tested negative. Personal protection measures were reinforced and made compulsory for the staff and actions were taken to raise farmers’ awareness of the biological risk of discharging animal carcasses as urban waste.

## Introduction

Q fever is a zoonotic disease caused by the obligate intracellular bacterium *Coxiella burnetii*. Domestic ruminants (cattle, sheep and goats) are the primary reservoirs of *C*. *burnetii*, and the bacteria are excreted at high numbers in birth products, as well as through milk, urine and faeces [1–3]. Placenta of infected sheep and goats can contain 10^9^ *C*. *burnetii*/ gram [4,5]. *C*. *burnetii* can survive in the environment for long periods, and human infection is mainly acquired by inhalation of aerosols containing infected dust particles from infection sources [6,7].

After an incubation period of 2–4 weeks, non-immune infected persons can develop acute Q fever that manifests as pneumonia, acute hepatitis or flu-like illness. Nevertheless, about 50–60% of cases remain asymptomatic. Both symptomatic and asymptomatic patients with acute Q fever, can progress to a chronic form that can lead to endocarditis in 1–5% of infected people [4,8].

Q fever is endemic in the Basque Country (northern Spain), and cases are voluntarily notifiable to the Basque System of Microbiological Information (*Sistema de Información Microbiológica* (SIM)) for an epidemiological assessment. In Bizkaia, the county where the outbreak reported herein occurred, the incidence of Q fever in recent years oscillated between 3.5 cases in 2010 to 2 cases in 2013 per 100,000 population. In the region (1,158,000 inhabitants), Q fever in humans has been historically associated with sheep since human cases appear in spring, coinciding with the end of the lambing season [9].

This study describes the clinical, epidemiological, diagnostic and environmental investigation of a Q fever outbreak occurred among workers of a solid waste-sorting plant in Bizkaia between February and April 2014, where 58.5% of the staff was affected.

## Materials and Methods

### Waste-sorting plant description

The plant, located in the municipality of Bilbao (Bizkaia, Basque Country, northern Spain), occupies 23,115 squared metres and has the capacity to handle up to 180,000 Tons of mixed urban residues per year. Residues are mechanically and biologically treated in order to recover the maximum percentage of recyclable material, achieve maximum heat recovery and stabilize the organic fraction. The plant is divided into different working areas, administration and 3 waste processing areas: receipt (waste is received, unloaded using a crane and bags opened), sorting (waste is separated into organic, inorganic, recyclable and non-usable residual waste) and biological treatment (biological components of the waste are dried and aerobically digested).

### Case presentation

On March 19, 2014, a patient was admitted through the emergency room of *Hospital Universitario de Cruces* with symptoms of atypical pneumonia. The patient reported similar symptoms among other employees from the waste-sorting plant where he worked. The hospital informed the local Epidemiology Surveillance Unit that contacted the waste-sorting plant. The company confirmed that the number of workers on sick leave due to flu-like illness had increased after February 22. Hospitals were then alerted and a higher index of suspicion was therefore required for workers of the plant. On March 25 the first case of confirmed *C*. *burnetii* infection was diagnosed.

### Epidemiological investigation

Once the outbreak was declared and *C*. *burnetii* identified as the causative agent, a multidisciplinary group that included microbiologists, veterinarians, occupational health technicians and epidemiologists was gathered to investigate the case, monitor the workers and design an epidemiological investigation that included the staff, the plant environment and ruminants grazing in the surroundings of the plant.

### Case definition

A confirmed human case was defined as a person who had worked at the plant between January 1, 2014, and May 23, 2014, and had a laboratory positive result (PCR *C*. *burnetii*-positive or specific antibody response—Phase II IgG or IgM), with or without clinical symptoms (fever, pneumonia and/or hepatitis). Workers with clinical symptoms but not confirmed by laboratory analyses were considered probable cases.

### Laboratory analyses

Presence of IgG antibodies to *C*. *burnetii* Phase II in human sera was investigated using a commercially available indirect immunofluorescence assay (IFA) (Vircell SL®, Spain), considering titres > = 1/256 as positive. Phase II IgM antibodies were detected by a commercial indirect ELISA (Vircell SL®, Spain), considering indexes 1.2–2 as weak positive and >2 as positive [10]. Diagnosis of chronic Q fever was carried out by IFA *C*. *burnetii* Phase I IgG antibody titres determination (Vircell SL®, Spain) considering titres > = 1/800 as positive. For molecular analyses 400 μl of whole human blood samples were submitted to DNA extraction using MagNA Pure Compact kit and tested by real-time PCR using a TaqMan probe specific for the transposon-like IS1111 repetitive region of *C*.*burnetii* [11]. Samples were analyzed in duplicates and qPCR reactions were considered positive when Ct values were below 40.

Animal blood samples were taken solely for the purpose of this study. After individual collection of blood into tubes without anticoagulant, sera were obtained by centrifugation and tested for the presence of antibodies against *C*. *burnetii* using a commercial indirect ELISA test (LSIVET Ruminant Milk/Serum Q Fever kit; Laboratoire Service International, Lissieu, France) as previously reported [12]. To identify active infection, ELISA positive sera were re-tested by means of the Complement Fixation test (CFT) following standard procedures [13].

Air (gelatine filters) and dust samples taken within the plant premises were collected as previously described [14], and analysed by real-time PCR (qPCR) [15]. Samples with a positive qPCR result and a cycle threshold (C_t_) value below 34 were submitted to Multiple Locus Variable number tandem-repeat Analysis (MLVA) to characterize the *C*. *burnetii* strain present. Two multicolour multiplex PCR assays were performed targeting six microsatellite markers containing either six (Ms27, Ms28 and Ms34) or seven (Ms23, Ms24 and Ms33) base pairs repeat units as described in detail elsewhere [16].

### Statistical analyses

Associations between personnel risk factors and working areas were analysed by Chi squared test (categorical variables) or by Student's *t*–test (numerical and dichotomous variables) using SPSS Statistic 21. Attack rates were assessed by Mantel-Haenszel Chi squared test using Epi Info 7 and Odds Ratios were assessed by exact test using Stata 12.1.

### Ethical considerations

This study did not require ethical approval since outbreaks are routinely investigated according to the Public Health services’ ethical guidelines to ensure patients safety. Blood samples were obtained by occupational health technicians involved in the study of the outbreak. TTEs were performed by radiologists from the Mutual Insurance Health Services. Written informed consent was obtained from the workers for blood sample collection and personal data collection following legal regulations (Ley Orgánica 15/1995). Some of the authors (epidemiologists and health technicians from OSALAN) directly interacted with patients to compile epidemiological data or to obtain blood samples from patients. Data analysis was performed on an anonymized dataset. Animal blood samples were taken solely for the purpose of this study by the veterinary practitioners in charge of the Official Sanitary Campaigns in the Basque Country, directed and supervised by the local Animal Health and Welfare Authority (Diputación Foral de Bizkaia) following Spanish ethical guidelines and animal welfare regulations (Real Decreto 53/2013). The collection of this material did not require the approval of the Ethics Committee for Animal Experimentation because they are considered routine veterinary practice. All herd owners had given an informed consent.

## Results

One week after the first case of pneumonia in a worker of the waste-sorting plant was declared, *C*. *burnetii* was identified by Phase II IgM detection in ELISA test (March 25). One week later (April 1) IgM antibodies appeared in another worker and two other cases were confirmed by qPCR, thus confirming *C*. *burnetii* as the causative agent of the outbreak. By then, 19 employees of the waste-sorting plant were on sick leave, 8 of them with a diagnosis of pneumonia.

On March 26, the waste-sorting plant was visited by the outbreak control team to gather information regarding plant characteristics, activities and possible risk practices. During the visit, the presence of ruminants grazing in the area surrounding the plant was observed. Besides, workers acknowledged that animal remains had been detected among the residues received for waste-sorting. An epidemiological investigation including workers, the grazing ruminants and the plant environment was designed. Activities at the plant were brought to a stop on April 10, but 42 workers remained in charge of the cleaning procedures until May 23.

**Fig 1:**
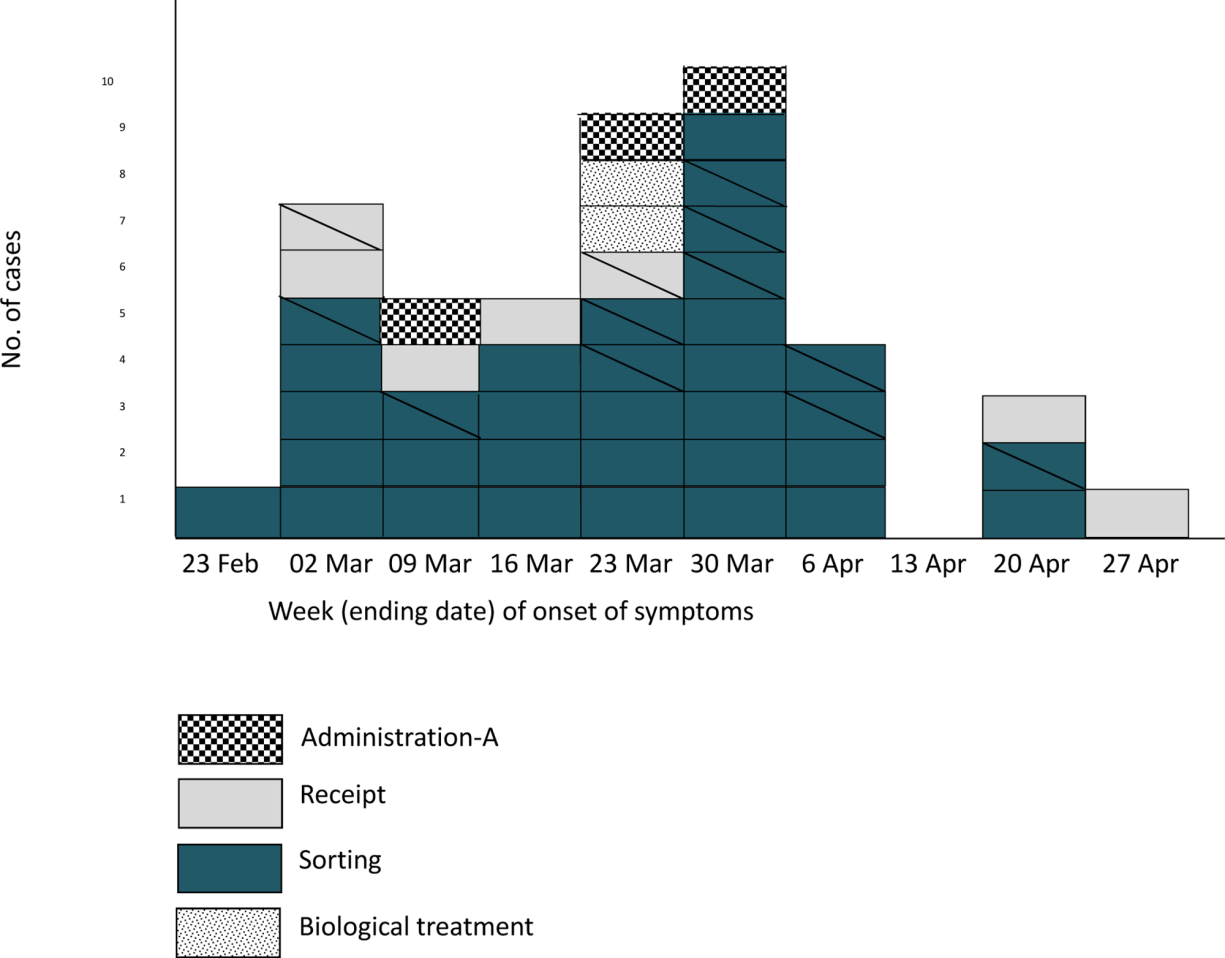
Epidemic curve based on the onset of symptoms for confirmed and probable cases by working area. Only 32 confirmed cases provided date of onset of symptoms. Probable cases (N = 12) are indicated by striped boxes.

**Fig 2:**
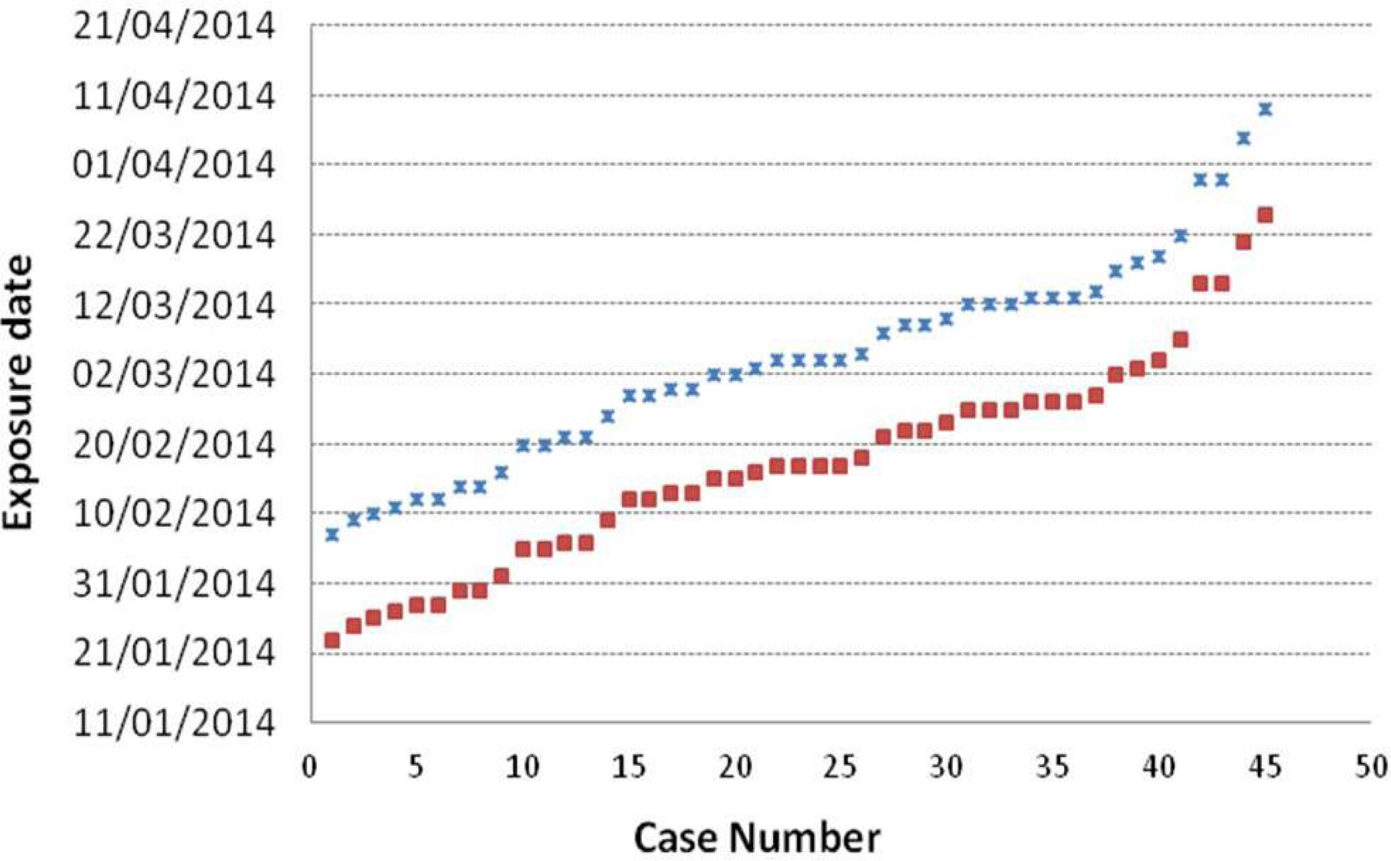
Estimated exposure date for Q fever infected workers. An incubation period of 2 weeks (crosses) or 4 weeks (squares) has been considered.

In April, a serological and epidemiological investigation was carried out. One hundred and six of the 125 workers (Table 1) who had access to the plant during the outbreak agreed to provide blood samples and fill a questionnaire to investigate risk factors of exposure (both at work and elsewhere). Workers were tested for the presence of *C*. *burnetii* Phase II IgG or IgM antibodies in sera collected 3 weeks apart (April 8–9 and April 29–30); PCR was also performed on blood samples from workers who had had symptoms within the three previous weeks. Fifty (47.2%) workers were identified as confirmed cases (4 workers confirmed by qPCR and serology, and 46 only by serology of the 106 tested), 33 of them with symptoms. Another 12 workers met the probable case definition (symptoms without laboratory confirmation), and the remainder 44 were regarded as non-cases (Table 1). Ten workers (9 confirmed and 1 probable) needed hospital admission with a median stay at hospital of 4 days. The epidemic curve representing the progression of illnesses onsetin confirmed and probable cases is shown in Fig 1. The first case occurred during the third week of February at the sorting area and the maximum incidence peak was reached on the last week of March. Workers at the biological treatment area of the plant were not affected till the fifth week after the outbreak started. Considering that incubation of Q fever takes 2 to 4 weeks, exposure was estimated to have extended from the end of January until April 10, when activities at the plant ended (Fig 2).

**Table 1:** Summary of the results obtained in the epidemiological questionnaire according to variables and case definition.

Epidemiological data		no. (%)			
		Confirmed cases (N = 50)	Probable cases (N = 12)	Non-case (N = 44)	Total workers (N = 106)
Sex	Male	41 (82.0)	9 (75.0)	35 (79.5)	85 (80.2)
	Female	9 (18.0)	3 (25.0)	9 (20.5)	21 (19.8)
Age	Median	40.5	39	41	40.5
	Min—Max	23–57	27–53	25–54	23–57
Symptoms	Any	33 (66.0)	12 (100.0)	7 (15.9)	52 (49.1)
	Fever	27 (54.0)	12 (100.0)	-	39 (36.8)
	Malaise	20 (40.0)	7 (58.3)	5 (11.4)	32 (30.2)
	Myalgia	11 (22.0)	6 (50.0)	4 (9.1)	21 (19.8)
	Headache	22 (44.0)	10(83.3)	6 (13.6)	38 (35.9)
	Pneumonia	13 (26.0)	3 (25.0)	-	16 (15.1)
Living in a rural setting		6 (12.0)	2 (16.7)	7 (15.9)	15 (14.2)
Contact with livestock (outside work)		3 (6.0)	1 (8.3)	7 (15.9)	11 (10.3)
Smoker		26 (52.0)	5 (41.7)	25 (56.8)	56 (52.8)
Use of respiratory protection	No	31 (62.0)	8 (66.7)	20 (45.4)	59 (55.7)
	Occasional	8 (16.0)	1(8.3)	4 (9.1)	13 (12.3)
	Yes	11 (22.0)	3 (25.0)	20 (45.5)	34 (32.1)

The male-to-female ratio of cases (confirmed and/or probable) did not differ significantly from the ratio of male-to-female employees. Likewise, no significant differences in incidence were observed according to age, smoking habits, living in a rural setting or being in contact with livestock outside work (Table 1). Confirmed cases were significantly associated (χ^2^ M-H 95% 4.87, p = 0.027) with workers who never wore respiratory masks compared to those who did. The association was also significant when considering confirmed and probable cases together (χ^2^M-H 95% 5.40, p = 0.020). Odds ratio calculations showed that probability of infection was 2.8 higher for workers who did never wear respiratory mask compared to those who used it (Table 2). Attack rates according to working area were analysed (Table 2). Thus, among workers who spend most of the working day at the waste processing areas of the plant (those ascribed to the receipt, sorting and biological treatment areas), the highest attack rate was detected at the receipt area (Table 2). A similar attack rate was observed for the personnel in Administration-area A, who worked at the offices but carried out control processes at the plant. Conversely, no single case was detected among the personnel ascribed to Administration-area B, the only workers who did not access the waste processing areas of the plant at any time (Table 2). Odds ratios for infection in relation to working area indicated that staff working at the receipt had 4.7 times higher probability to acquire infection than those working in Administration; odds ratio for those working at the sorting area was 3.9. However, differences in attack rate according to the area where workers carried out their activities were not significant (Table 2).

**Table 2:** Attack rates and odds ratios (OR) according to working area, use of respiratory protection and case definition.

		no. of Workers	Confirmed cases			Confirmed + Probable cases		
			Cases	Attack rate %	OR (95% CI)	Cases	Attack rate %	OR (95% CI)
Working area	Receipt	18	10	55.5	3.9 [0.7–21.1]	12	66.6	4.7 [0.9–24.8]
	Sorting	72	35	48.6	3 [0.7–12.8]	45	62.5	3.9 [0.9–16.3]
	Biological treatment	6	2	33.3	1.2 [0.1–10.2]	2	33.3	1.2 [0.1–10.2]
	Administration-A	5	3	60.0	1^a^	3	60.0	1 ^a^
	Administration-B	5	0	NA		0	NA	
	TOTAL	106	50	47.2		62	58.4	
Respiratory protection	No	59	20	52.5	2.8 [1.1–7.1]	39	66.1	2.8 [1.2–6.6]
	Occasional	13	4	61.5	3.6 [0.9–14.9]	9	69.2	3.2 [0.8–12.5]
	Yes	34	20	32.4	1^b^	14	41.2	1 ^b^

NA, non-applicable

^a^ Staff working in Administration (A+B) was used as reference group for working area.

^b^ Staff using respiratory protection was used as reference group for respiratory protection.

A follow-up of patients was carried out to assess possible evolution to chronic Q fever. Risk factors for development of chronic Q fever were investigated and a transthoracic echocardiogram (TTE) was performed in all patients with confirmed (49, all but one who refused to be included in the study) and probable infection (12). Following routine procedures, patients with normal TTE and no risk factors were discharged; patients with a valvulopathy or risk factors were serologically monitored (IFA *C*. *burnetii* Phase I IgG antibody titres) every three months during a year [10,17]. Here, TTE was normal in the 61 cases studied. Neither lesions in endocardium nor valvular heart disease were observed. Phase I monitoring was carried out in two patients with diabetes mellitus and sleep apnea, respectively, and in both cases IgG titers were below 1/126. As at the date of writing this manuscript (July 2015), no cases of chronic Q fever infection were detected.

Animal and environmental investigations started at the beginning of April. Thus, between April 3 and April 10 a total of 81 goats from 8 farms that grazed in fields nearby the plant were sampled. Presence of antibodies against *C*. *burnetii* was detected in only one farm where 6 of 27 goats (22%) were seropositive by ELISA test but negative by CFT, suggesting that infection was not active. Environmental samples (surface dust and aerosols) were collected within the plant premises at three different time points: one week after the first case was confirmed (April 4), before cleaning and disinfection of the premises (May 9) and 10 days after disinfection (June 25). Thirty-nine samples of dust and 1–4 air samples were collected per sampling. All aerosol samples were qPCR negative, but *C*. *burnetii* DNA was detected in a high percentage of dust samples in the first (56%) and second visit (51%) (Table 3). After cleaning and disinfection (third visit) dust samples were all negative. A selection of qPCR-positive samples with a C_t_ value below 34 (n = 7) were genotyped by MLVA. A complete genotype (genotype AE) was only obtained for the sample with the lowest C_t_ (25), which corresponded to a sample taken from the waste sorting area during the first visit.

**Table 3:** Progression of *C*. *burnetii* DNA detection in the environment before (1^st^ and 2^nd^ samplings) and after cleaning and disinfection (3^rd^ visit).

Type of sample	Plant working area	no. Positive / Analysed (%)		
		1^st^ sampling (04/04/14)	2^nd^ sampling (09/05/14)	3^rd^ sampling (25/06/14)
Aerosols	Receipt	0/1 (0.0)	0/1 (0.0)	
	Sorting	0/1 (0.0)	0/1 (0.0)	0/1^c^ (0.0)
	Biological treatment	0/1 (0.0)	0/2 (0.0)	
	Total aerosol samples	0/3 (0.0)	0/4 (0.0)	0/1 (0.0)
Dust	Sorting	15/25 (60.0)	12/25 (48.0)	0/25 (0.0)
	Biological treatment	6/11 (54.5)	7/11 (63.6)	0/11 (0.0)
	Common areas	1/3 (33.3)	1/3 (33.3)	0/3 (0.0)
	Total dust samples	22/39^a^ (56.4)	20/39 ^b^ (51.3)	0/39 (0.0)

^a^ Positive dust samples from the first visit had Ct values of 25 (1 sample), 32 (1 sample) and ≥34 (the remaining 20 samples).

^b^ In the second visit, all positive dust samples (20) had Ct≥34.

^c^ In the 3^rd^ visit no electricity was available due to previous cleaning and disinfection activities. Only 1 sample could be taken.

Cleaning procedures started on April 10 (when activities at the plant ended) with the emptying of the plant, which was carried out by 42 workers of the plant; cleaning and disinfection was later performed by a specialized company. The disinfection protocol included a first stage of contact disinfection by spraying a balanced, stabilized blend of peroxygen compounds, surfactant, organic acids, and inorganic buffer in a 1:100 dilution (Virkon S, Dupont Ibérica S.L., Barcelona, Spain), and a second aerial disinfection by nebulization with glutaraldehyde (Total Shock SR, José Collado S.A., Barcelona, Spain) and paraformaldehyde tablets (Formaster, Zoetis, Spain). Other outbreak control measures implemented after the first visit to the plant included the instructions to remove any remains of animals from the residues at receipt as soon as detected, and the reinforcement of all measures of personal protection, including the compulsory use of respiratory masks (filtering face pieces class 2—FFP2 or above-, as established by European standard EN149:2001) for all the staff entering the waste processing areas of the plant, adequate clothing, and eye protection goggles for job descriptions with risk of splashes. Cleaning operations were tightened and included daily cleaning of common areas and, industrial cleaning of work clothing. The city council and farmers were reminded of the banning of home slaughtering (Real Decreto 2018/2000) and the discharge of animal carcasses as urban waste.

## Discussion

Certain occupations, mainly those that involve contact with animals, are associated with increased risk for exposure to *C*. *burnetii*. Multiple Q fever outbreaks have been reported among workers in slaughterhouses, veterinarians, farmers, shearers, or livestock transport drivers [4,8,18]. Only a few localized outbreaks have been reported at work settings with apparently no occupational-associated risk [19,20], and to our knowledge this is the first report of an outbreak among workers of a waste-sorting plant. The Q fever outbreak described herein affected 58.5% of the investigated workers of the plant. Still, number of cases might have been underestimated since only those who had been working for more than a week in the plant were monitored, and during the course of the outbreak, temporal recruits were necessary to cover up for the personnel on sick-leave. Only 32.0% of the confirmed cases were asymptomatic, which is lower than that reported in other outbreaks [19–21]. Although Q fever illness is more commonly reported in males than females [10], it might be partly explained by sex-associated occupational exposures. Here, the attack rate in this outbreak did not differ between male and female workers, probably due to the similar exposure to the pathogen in their workplaces, a situation also observed in other outbreak investigations [20]. However, gender in combination with age has been shown to affect the risk of developing acute Q-fever [22], with the gender difference may be due to the protective effect of 17β-estradiol in females [23].

The detection of *C*. *burnetii* DNA in dust samples collected from the surfaces of the plant facilities confirmed the exposure of workers to the infection inside the plant. The temporal distribution pattern of the infection among workers at the different sections fit in well with the layout of the plant. The first case occurred at the sorting area. In fact, the dust sample with the highest concentration of *C*. *burnetii* DNA (lowest C_t_ value) corresponded to a sample taken from the waste sorting area during the first sampling. Dust-generating tasks like the mechanical process of sorting waste through the belts circuit would favour the circulation of *C*. *burnetii* laden dust that infected susceptible workers by inhalation of contaminated aerosols, particularly if no respiratory protection masks were worn. Infection then progressed towards the biological treatment area, where infection was apparent 5 weeks after the first case. It was then, during the second half of March when infection incidence reached its highest peak. Accordingly, a constantly high percentage of dust samples remained positive at the first two samplings (April 4 and May 9), in agreement with the long lasting resistance of *C*. *burnetii* in the environment [18,24]. However, once cleaning started, and dust was removed and surfaces disinfected, no *C*. *burnetii* DNA was detected, thus demonstrating the success of the cleaning and disinfection procedures implemented. Therefore, workers were exposed to *C*. *burnetii* during *ca*. 65 days, and the only employees who were not affected were those working at the Administration with no access to the waste processing areas of the plant (Administration-area B).

The outbreak happened at the beginning of spring, which is the main lambing season for small ruminants in the region. However, laboratory tests performed on animals grazing in the fields surrounding the plant did not show active infection in the nearby farms, thus ruling out their role as source of infection. Still, this seasonal association suggested that highly infected tissues from sheep or goats might have arrived at the waste-sorting plant sometime at the end of January 2014. In fact, in January 2014 the volume of waste received at the plant had increased, and in addition to urban waste, waste from rural areas started to be processed.

Genotyping of *C*. *burnetii* isolated from clinical and environmental samples has been helpful in identifying the strains involved in active Q fever episodes and to determine the ruminant sources involved in Q fever outbreaks [25]. In the current study, *C*. *burnetii* MLVA genotype AE was identified in one dust sample. This genotype had been previously identified in Bizkaia, both in a goat placenta [16] and in a bulk-tank milk sample from dairy cattle [26]. Unfortunately, human DNA samples from this outbreak could not be genotyped due to their high qPCR Ct value (C_t_>37) [27]. As far as we know, genotype AE has not yet been found in human clinical samples, but this work suggests that this genotype was the cause of this Q fever outbreak, with pneumonia as the main symptom observed in patients.

These findings underscore the need to implement adequate biosecurity measures to reduce risk of occupational exposure to zoonotic pathogens that might be present in waste material, especially if animal tissues potentially infected are incorrectly disposed as urban waste. After the outbreak, personal protection measures against biological hazards were reinforced and made compulsory for the staff of the plant and actions were taken to raise farmers’ awareness of the biological risk associated to an inadequate discharge of animal carcasses. Population in general and farmers in particular should be fully conscious of the consequences that bad practices could have on Public Health. Compliance with these control measures is particularly crucial at lambing season when risk of infection is highest.
